# Unilateral Conductive Hearing Loss Disrupts the Developmental Refinement of Binaural Processing in the Rat Primary Auditory Cortex

**DOI:** 10.3389/fnins.2021.762337

**Published:** 2021-11-19

**Authors:** Jing Liu, Xinyi Huang, Jiping Zhang

**Affiliations:** Key Laboratory of Brain Functional Genomics, Ministry of Education, NYU–ECNU Institute of Brain and Cognitive Science at NYU Shanghai, School of Life Sciences, East China Normal University, Shanghai, China

**Keywords:** unilateral conductive hearing loss, binaural processing, binaural interaction, auditory cortex, interaural level differences, rats

## Abstract

Binaural hearing is critically important for the perception of sound spatial locations. The primary auditory cortex (AI) has been demonstrated to be necessary for sound localization. However, after hearing onset, how the processing of binaural cues by AI neurons develops, and how the binaural processing of AI neurons is affected by reversible unilateral conductive hearing loss (RUCHL), are not fully elucidated. Here, we determined the binaural processing of AI neurons in four groups of rats: postnatal day (P) 14–18 rats, P19–30 rats, P57–70 adult rats, and RUCHL rats (P57–70) with RUCHL during P14–30. We recorded the responses of AI neurons to both monaural and binaural stimuli with variations in interaural level differences (ILDs) and average binaural levels. We found that the monaural response types, the binaural interaction types, and the distributions of the best ILDs of AI neurons in P14–18 rats are already adult-like. However, after hearing onset, there exist developmental refinements in the binaural processing of AI neurons, which are exhibited by the increase in the degree of binaural interaction, and the increase in the sensitivity and selectivity to ILDs. RUCHL during early hearing development affects monaural response types, decreases the degree of binaural interactions, and decreases both the selectivity and sensitivity to ILDs of AI neurons in adulthood. These new evidences help us to understand the refinements and plasticity in the binaural processing of AI neurons during hearing development, and might enhance our understanding in the neuronal mechanism of developmental changes in auditory spatial perception.

## Introduction

The central auditory system receives, integrates, and analyses inputs from the two ears. This binaural processing contributes to localizing the sound source (Middlebrooks, 2015), separating the target sound from competing noisy background (Middlebrooks and Waters, 2020), and improving speech perception in noise (Hawley et al., 2004). The perception of acoustic space in humans exhibits developmental changes in sound localization accuracy and auditory spatial discrimination (Van Deun et al., 2009; Kuhnle et al., 2013), and it develops from an initially imprecise representation of spatial positions in infants and young children to a concise representation in young adults (Kuhnle et al., 2013; Freigang et al., 2015). In addition, the sensitivity to binaural cues in human is evident early in life (Bundy, 1980) and reaches adult-like behavior by 4–5 years of age (Van Deun et al., 2009). It is generally believed that the binaural cues for the perception of the sound-source location in horizontal plane are the interaural time differences (ITDs) and interaural level differences (ILDs), and the ILDs provide the major cue for the horizontal location of high-frequency sounds (Middlebrooks, 2015). Animal studies have shown the importance of binaural processing in sound localization, e.g., the sound localization accuracy can be disrupted when one ear was occluded with an earplug (Potash and Kelly, 1980; Keating et al., 2015). However, the neural mechanism for the normal development of binaural processing is still not fully understood.

The primary auditory cortex (AI) has been demonstrated to be necessary for binaural processing. Lesion or inactivation of AI leads to sound localization deficits (Malhotra and Lomber, 2007; Nodal et al., 2010). AI neurons have been shown to be sensitivity to sound-source azimuth in cats (Samson et al., 1994), monkeys (Woods et al., 2006), bats (Razak, 2011), ferrets (Wood et al., 2019), and rats (Yao et al., 2013; Gao et al., 2018; Wang et al., 2019). The spatial sensitivity of AI neurons to sound-source azimuth largely depends on binaural processing. Abnormal inputs from the two ears disrupt the tuning of AI neurons to sound-source azimuth (Samson et al., 1994; Wang et al., 2019). The binaural cues are first computed in the superior olive complex (Grothe et al., 2010), and the ILDs are further processed in the inferior colliculus (Semple and Kitzes, 1987) and auditory cortex (Zhang et al., 2004). Based on the spiking responses of AI neurons to monaural and binaural stimuli at corresponding stimulus levels, the integrations of the inputs from both ears are often categorized into facilitatory, inhibitory, and mixed binaural interactions (Irvine et al., 1996; Zhang et al., 2004). These studies on the binaural processing in AI focused on the adult animals. Studies using auditory evoked potentials to measure the binaural interaction of newborn infants have demonstrated immature binaural interactions at brainstem (Cone-Wesson et al., 1997) and auditory cortex (Nemeth et al., 2015). However, at single neuron level, how the binaural processing in AI develops after hearing onset is not fully elucidated.

After the onset of hearing, the auditory cortex undergoes developmental refinements in the tonotopic map of sound frequency (Zhang et al., 2001), and in the spectral and temporal response selectivity (Chang et al., 2005; Zhao et al., 2015; Cai et al., 2018). The role of inhibition contributes to the progressive maturation in frequency tuning (Chang et al., 2005) and temporal processing (Cai et al., 2018). The pace of cortical synaptic receptive field development is set by progressive, experience-dependent refinement of intracortical inhibition (Dorrn et al., 2010) or a fine adjustment of excitatory input strength (Sun et al., 2010). As the sound localization ability improves from infants to young adults, we hypothesize that the binaural processing which contributes to sound localization might also undergo a progressive refinement in AI at single neuron level after hearing onset.

Humans with unilateral conductive hearing loss (UCHL), such as congenital UCHL and otitis media with effusion in early childhood, show persistent binaural hearing deficits after corrective surgery (Pillsbury et al., 1991; Wilmington et al., 1994). Neurophysiology studies have shown that chronic UCHL in rats from early onset of hearing disrupts the normal spatial azimuth tuning of AI neurons in adulthood (Wang et al., 2019). Monaural deprivation in young rats enhances the responsiveness of inputs from the developmentally opened ipsilateral ear in AI and disrupts the binaural integration of ILD (Popescu and Polley, 2010). In addition, brief UCHL at young age disrupts the normal coregistration of interaural frequency tuning and ILD sensitivity in the mice AI (Polley et al., 2013). These studies have enhanced our understanding of how UCHL at young age induces the experience-dependent plasticity in AI. Otitis media with effusion often induces UCHL in human infants (Hogan et al., 1997), and the binaural hearing abilities was not completely restored even if the hearing threshold returned to normal after corrective surgery for the UCHL (Pillsbury et al., 1991). Until now, whether and how the reversible UCHL (RUCHL) at young age affects the developmental refinement of binaural processing in the adult AI is not fully understood.

In the present study, we first investigated the developmental refinement of binaural processing in AI by determining and comparing the monaural response types and the binaural processing properties (i.e., the binaural interaction types, the degree of binaural interactions, the sensitivity and selectivity to ILDs) of AI neurons among different age groups of rats with normal hearing development. We then studied the effects of RUCHL at young age on the binaural processing of AI neurons in adulthood by comparing the binaural response properties of AI neurons between the normal developing adult rats and the RUCHL rats in adulthood. We have demonstrated that there exists a developmental refinement of binaural processing in AI after hearing onset, and that RUCHL at young age disrupts the developmental refinement of binaural processing in AI.

## Materials and Methods

### Animals and Animal Groups

Four groups of Sprague–Dawley rats were used: (1) group 1, postnatal day (P) 14–18 rats (*n* = 45), the ages of these rats were within 1 week after hearing onset (usually at P12); (2) group 2, P19–P30 rats (*n* = 53); (3) group 3, P57–P70 adult rats (*n* = 47); (4) group 4, RUCHL rats (*n* = 60, P57–70) with UCHL only during P14–30. Rats in groups 1–3 had normal binaural hearing development. For convenience, we randomly picked the rats with different ages and assigned them to the four groups from P14, i.e., the P14–18 group, the P19–30 group, the adult group, and the RUCHL group, respectively. The adult group was also used as the control to study the effects of RUCHL at young age on the binaural processing of AI neurons in adulthood. The rats were bred in-house from Sprague–Dawley breeding pairs purchased from Shanghai Jie Si Jie Laboratory Animal Co., Ltd., (Shanghai, China). The rat pups were raised with their parents until P26. All rats had free access to food and water, and were reared in the housing environments (20–24°C temperature) with 12-h light–dark cycles.

For the rats in the RUCHL group, unilateral middle ear poloxamer hydrogel injections adapted from a previous study were made to induce RUCHL at young age (Polley et al., 2013). Briefly, after the P14 rats were anesthetized by Nembutal (50 mg/kg, i.p.), a slit in the right tragus was made to better visualize the tympanic membrane in the right ear. A small hole was made in the pars flaccid to allow the injection of poloxamer 407 solutions through a glass capillary with about 15 μm tip in diameter. The blunt end of the glass capillary was attached to the syringe infusion set, and the syringe was filled with a 30% (w/w) solutions of poloxamer 407 and blue dye. About 10 μl poloxamer solutions (around 4°C) were injected to the middle ear to fill the middle-ear cavity under an operating microscope. Additional two injections of 5 μl poloxamer 407 solutions were done at P16 and P18, respectively. The poloxamer 407 solutions rapidly transitioned to gels in the middle ear cavity at body temperature after injection, which induced a conductive hearing loss at the injected ear. The poloxamer 407 gels spontaneously dissolved through hydrolysis several days later, and the thresholds of auditory brainstem response (ABR) were fully resolved 14–15 days after the initial poloxamer injection (Polley et al., 2013). In this way, this method provides us a convenient way to induce RUCHL in rats. We determined the hearing threshold of each ear by measuring the ABR wave I threshold. For a small number of rats (*n* = 11), ABR measurements were conducted for each ear at P14–P30 with an interval of 2 days. For all the rats in the RUCHL group, ABR measurements were conducted for each ear on the days of injection and P30. At P30, the hearing threshold of the injected ear was considered to be recovered to normal levels if the ABR threshold difference between the injected ear and the normal control ear of a rat was less than 5 dB. These rats were raised until adulthood, and they constituted the RUCHL group of rats.

### Acoustical Stimulus Presentation System

Acoustic stimulus presentation were performed through TDT System 3 hardware and software (Tucker-Davis Technologies, United States) controlled by a PC. The hardware for acoustic stimulus presentation includes a multifunction processor (RX6-A5), a stereo power amplifier (SA1), and two multi-field magnetic speakers (MF1). All acoustic stimuli were delivered to ears via a close-field system. The speakers (MF1) were incorporated internal parabolic cones and coupled to the ears through 9.5-cm-long PVC plastic tubes (1/16 inch ID, 1/8 inch OD, and 1/32 inch wall thickness) leading to the ear canal. Adaptable plastic tubes were used to couple the ear canals of infant rats when necessary. The end of each tube was about 5 mm from the tympanic membrane. The output of each MF1 speaker was calibrated from 2.0 to 44.0 kHz (sampling rate, 100 kHz) using a 1/4-in. condenser microphone (model 7016; ACO Pacific Inc.). The calibration data were stored in a computer for obtaining the desired sound pressure levels in decibel (dB SPLs, re: 20 μPa) within the calibrated frequency range.

### Auditory Brainstem Response Measurement

The procedure for ABR measurement was similar to that described in our previous study (Wang et al., 2019). Briefly, rats were anesthetized with Nembutal (50 mg/kg, i.p.) and then placed in a stereotaxic frame in a double-walled sound-proof room. The acoustic signal was present from the MF1 speaker coupled to the ear. The subdermal needle electrodes (Rochester Electro-Medical, Inc., United States), headstage (RA4LI), preamplifier (RA4PA), and RX5A2 processor were used to record ABR signals. The electrodes were placed subcutaneously at the vertex (active), the mastoid ipsilateral to the acoustic signal source (reference), and the tail of the rats (ground). The ABR thresholds were measured independently for both ears with tone bursts (5 ms duration, 0.5 ms cosine ramps, 21 Hz repetition rate). The tone bursts were 4.0–36.0 kHz in frequency with 4.0-kHz increments and were 80 to 15 dB SPL in level with 5-dB decrements (or 2-dB steps at near ABR wave I threshold by visual inspections). The ABR signals were bandpass filtered (0.3–3 kHz), averaged from 512 stimulus pairs, and analyzed in BioSigRP software. The ABR Wave I threshold was defined as the lowest sound level that could reliably produce an acoustic stimulus-evoked peak which followed the progressive trend for decreasing amplitude and increasing latency obtained over the range of tested sound levels (Popescu and Polley, 2010). We determined the ABR Wave I threshold by visual inspections of ABR wave data, and by using a statistical measure, i.e., the lowest sound level that evoked a wave I with the peak-to-peak amplitude greater than 2 SDs of the background activity.

### Animal Surgery and Single-Unit Recording in Primary Auditory Cortex

Rats were anesthetized by urethane (1.5 g/kg, i.p.) before surgery and were given an injection of atropine sulfate subcutaneously (0.01 mg/kg) to reduce bronchial secretions. Body temperature was monitored and maintained at 37.5°C by an animal temperature regulator. The surgical procedures were similar to that described in our previous studies (Wang et al., 2019). Briefly, the trachea of the rat was cannulated to allow unobstructed respiration, and then a midline skin incision was made on the rat head to allow the exposure of the dorsal and temporal skull. A nail (4 cm long) was attached to the dorsal surface of the skulls with 502 super glue and dental cement. The rat was then fixed to a head holder through the nail. A small hole was made over the left auditory cortex. The dura was removed, and the exposed cortex was kept moist by warm saline.

The neurophysiologic recording was conducted in a sound-proof room. Glass electrodes (filled with 2 M NaCl, 1.0–2.0 MΩ impedance) were advanced orthogonally to the pial surface of AI by a remotely controlled microdrive (SM-21; Narishige, Japan). The recording depth of AI neurons was within the range of 300–900 μm under the pial surface. Action potentials were recorded, amplified (× 1,000), and band pass filtered (0.3–3.0 kHz) by a DAM80 amplifier (WPI, United States), and then fed into a pre-amplifier RA8GA, a RX5A2, and a PC for online and offline data processing. The signal was also monitored on an oscilloscope (TDS 2024, United States) and an audio monitor.

The responses of rat AI neurons were determined by presenting tone bursts varied with frequencies and levels. The frequencies were varied within the range of 4.0–44.0 kHz with 1-kHz increments, and the levels were varied within the range of 0–80 dB SPL with 10-dB steps. The tonal stimuli (50 ms duration including 1.5 ms rise/fall time) were monaurally presented to either ear or binaurally presented to the two ears in dichotic conditions. Absolute stimulus levels (dB SPL) and ILDs (dB) were set by computer through OpenEX software (TDT system 3). The interstimulus interval was 800 ms. The searching stimuli were presented monaurally to either ear and binaurally with equal levels at both ears. Single units were identified by the criteria of equal spike height, constant wave form, and significant signal-to-noise ratio.

### Data Analysis and Binaural Interaction Classification

**FIGURE 1:**
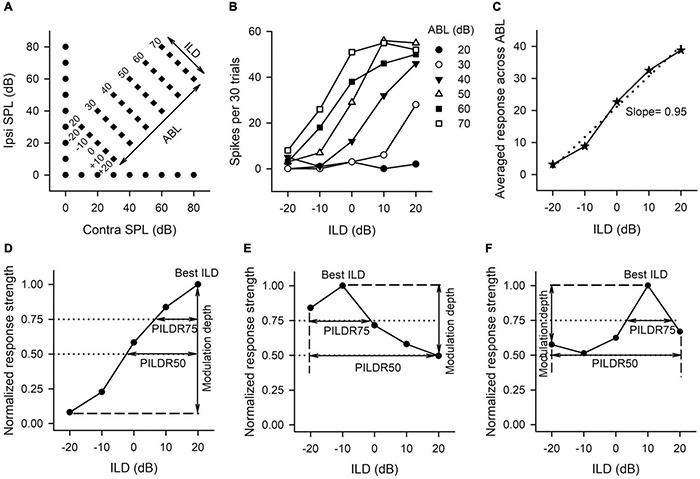
The sound stimulus paradigm and the method to determine the neuronal sensitivity and selectivity to ILDs from the ILD response functions of AI neurons. **(A)** The sound stimulus paradigm. The monaural stimuli (filled circles) and the binaural stimuli (filled diamonds) are designated in terms of contralateral (contra) and ipsilateral (ipsi) sound pressure levels in dB (dB SPL). The binaural stimulus can also be shown in terms of interaural level difference (ILD) and average binaural level (ABL). Positive ILDs favor the contralateral ear and negative ILDs favor the ipsilateral ear. **(B)** The ILD response functions of an AI neuron determined at different ABLs. **(C)** The averaged ILD response function across ABLs from the data in panel **(B)** (solid line) and the linear curve fitting function (dotted line). The slope value of this linear function was used as a measure of neuronal sensitivity to ILDs. **(D–F)** The method to determine the modulation depth, the preferred ILD range (PILDR75, PILDR50), and the best ILDs from the normalized ILD response functions. See texts in section “MATERIALS AND METHODS” for details.

Once a single neuron was identified, the characteristic frequency (CF; the frequency at which the neuron showed the lowest response threshold) was determined by presenting various frequency-level combinations in the audiovisually determined frequency-level response range. The rat AI was identified based on the unique rostral-to-caudal tonotopy (Doron et al., 2002) and short-latency responses to sound stimuli in the auditory cortex. The monaural rate-level functions were determined by presenting CF tonal stimuli (repeated 30 times) to the contralateral ear or the ipsilateral ear from 0 to 80 dB SPL in 10 dB increments (Figure 1A, filled circles). Then a matrix of binaural stimuli at CF was presented in which the ILDs and the average binaural levels (ABLs) were varied systematically in dichotic conditions (Figure 1A, filled diamonds). The ILDs were varied from −20 to + 20 dB in 10-dB steps, and the positive ILDs indicate greater sound levels in the contralateral (right) ear. The ABL was defined as the sum (in dB) of the contralateral and ipsilateral sound levels divided by 2. At each ILD, ABLs were varied from 20 to 70 in 10-dB steps. A binaural stimulus can be described in terms of its ILD and ABL, or in terms of the levels at the contralateral and the ipsilateral ears (Figure 1A). The binaural stimulus matrix included 30 binaural level combinations (repeated 30 times each).

The sensitivity and selectivity of AI neurons to ILDs were evaluated from the ILD response functions, i.e., spike counts versus ILD functions at various ABLs (Figure 1B), and averaged ILD response functions across ABLs (Figure 1C). A linear curve fitting was performed to the averaged ILD response function (Figure 1C, dotted line), and the slope value of this linear function was used as a measure of neuronal sensitivity to ILDs. A greater absolute slope value indicates greater sensitivity to ILD. We then normalized the response strength relative to the maximum spike counts in the averaged ILD response function, and the maximum response strength was 1.0. In the normalized ILD response functions (Figures 1D–F), the best ILD was defined as the ILD at which the normalized response strength was 1.0, and the modulation depth was defined as the differences in the normalized response strength between the maximum value and the minimum value (Figures 1D–F, vertical lines with double arrow head). A greater modulation depth indicates a greater sensitivity to ILDs. The selectivity of AI neurons to ILDs was assessed from normalized ILD response functions by determining the preferred ILD range (PILDR) over which the normalized response strength was 0.75 (PILDR75) and 0.50 (PILDR50) (Figures 1D–F). In the bottom row of Figure 1, the dotted lines show the normalized responses strength at 0.75 and 0.5, respectively, and the horizontal line with the double arrow show the PILDR75 and PILDR50 (Figures 1D–F). If the dotted line intersects with only one side of the normalized ILD response function, the PILDR was defined as the ILD range from the intersect of the dotted line with the normalized ILD response function to the contralateral limit (i.e., + 20 dB ILD, Figure 1D) or the ipsilateral limit (i.e., −20 dB ILD, Figure 1E). If the dotted line intersects with both sides of the normalized ILD response function, the PILDR was the ILD range between the two intersects (Figure 1F, PILDR75). In contrast, if the dotted line did not intersect with either side of the normalized ILD response function, the PILDR was defined as the ILD range from −20 to + 20 dB (Figure 1F, PILDR50). For the few instances where the ILD function has multiple PILDR75s or PILDR50s, the PILDR was defined as the sum of the extent of the PILDRs. The location of the PILDR indicates ILD preference, and the width of the PILDR was used as a measure of ILD selectivity. A narrower PILDR indicates a greater ILD selectivity. Using the same idea, for each neuron, we also analyzed the PILDR75 and PILDR50 from the ILD response function at each ABL to determine the ILD selectivity at different ABLs.

**FIGURE 2:**
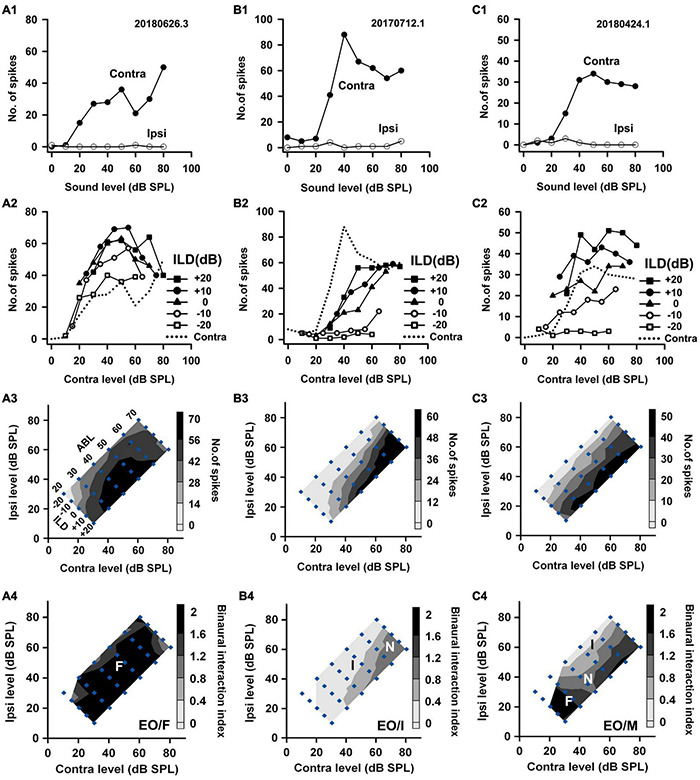
The responses of three EO neurons to both monaural and binaural stimuli. The data shown in each column are from one neuron. **(A1–C1)** The monaural responses of the neurons to stimuli from the contralateral (contra) ear and the ipsilateral (ipsi) ear. **(A2–C2)** The binaural responses of the neurons to stimuli in the binaural stimulus matrix plotted as a function of contralateral levels and at different ILDs. For comparison, the monaural contralateral response functions are shown in dotted lines. **(A3–C3)** The binaural response contours plotted at different contralateral and ipsilateral levels within the binaural stimulus matrix. Filled diamonds represent binaural stimulus conditions, and the stimuli are also shown in ABL vs. ILD in panel **(A3)**. **(A4–C4)** The contour plots of the binaural interaction index within the binaural stimulus matrix. F, I, and N: facilitatory, inhibitory, and no binaural interaction in the contour plots, respectively. The binaural response type of a neuron was classified as mixed (M) if both facilitatory and inhibitory binaural interactions occurred within the binaural stimulus matrix (e.g., neuron C). The three neurons were categorized as EO/F, EO/I, and EO/M, respectively.

**FIGURE 3:**
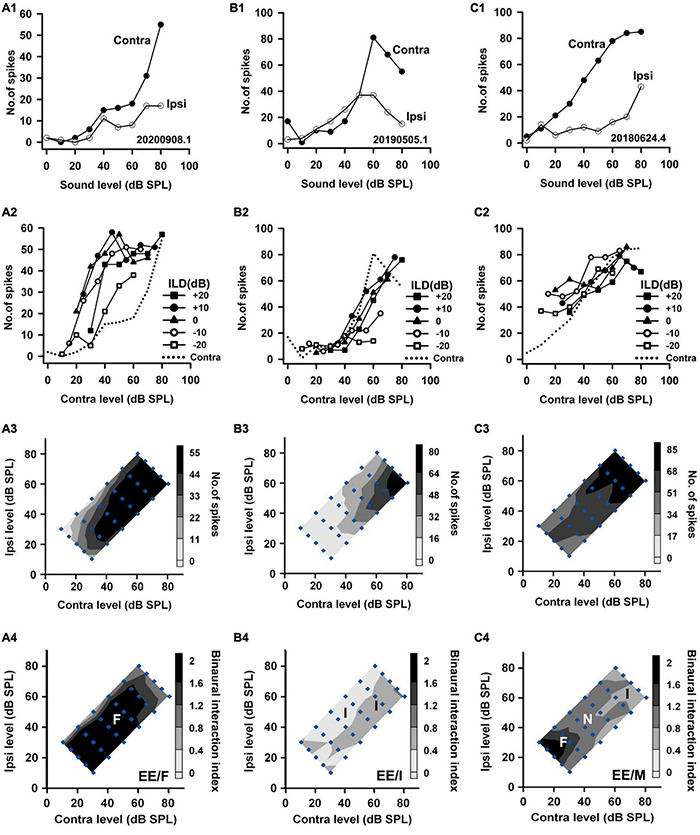
The responses of three EE neurons to both monaural and binaural stimuli. The data shown in each column are from one neuron. The legends for the panels in the four rows are similar to those in Figure 2. According to monaural responses and the binaural interaction behaviors in the binaural stimulus matrix, the three neurons were categorized as EE/F, EE/I, and EE/M, respectively.

**FIGURE 4:**
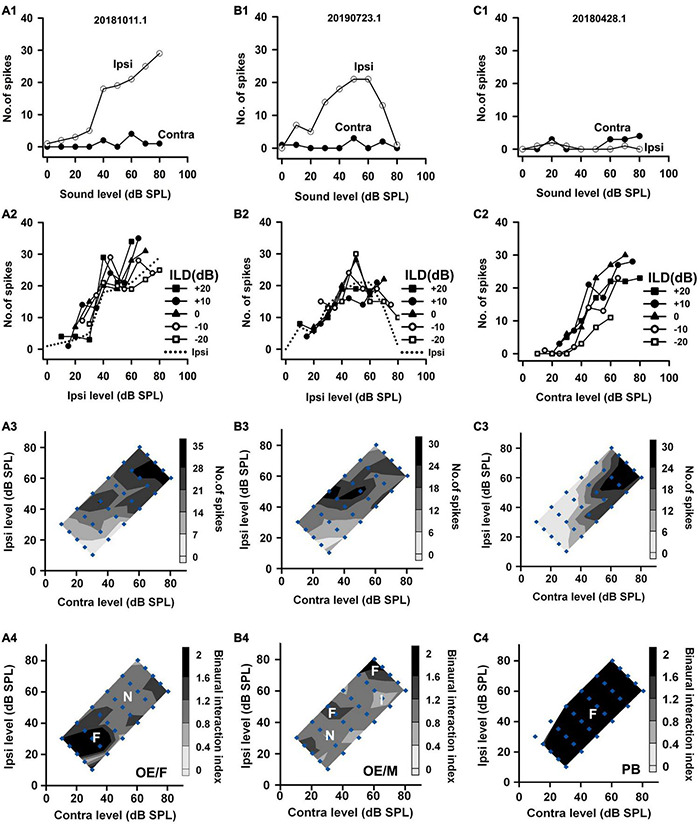
The responses of two OE neurons and one PB neuron to both monaural stimuli and binaural stimuli. The data shown in each column are from one neuron. The legends for the four rows are similar to those in Figure 2. The three neurons were categorized as OE/F, OE/M, and PB, respectively.

We categorized the binaural interaction of rat AI neurons following a previous classification scheme (Zhang et al., 2004). Rat AI neurons were first classified as EE, EO, OE, and PB types according to their monaural response properties (Figures 2–4): EO if the neuron was responsive to monaural stimulation in the contralateral ear but not in the ipsilateral ear (Figure 2); EE if it was driven by monaural stimulation of either ear (Figure 3); OE if the neuron responded to monaural stimulation in the ipsilateral ear but not in the contralateral ear (Figure 4); and PB (i.e., predominantly binaural) if the neuron did not respond or responded very weakly to monaural stimulation of either ear, but did respond strongly to binaural stimulation (Figure 4). Neurons within the category of each monaural response type were further classified according to their binaural interaction behavior within the binaural stimulus matrix. To quantify the degree of binaural interactions, we computed a binaural interaction index by dividing the response in spike counts to a binaural stimulus by the sum of the monaural responses in spike counts at corresponding monaural stimulus levels. In the present study, all of the recorded neurons showed onset responses. The responses to sound stimuli in spike counts were determined over the sound duration. Similar to the scheme used to classify the binaural interaction type (Zhang et al., 2004), a binaural interaction evoked by a binaural stimulus in the matrix was considered as facilitatory (F) if the binaural interaction index was greater than 1.2, inhibitory (I) if the binaural interaction index was less than 0.8, or no interaction (N) if the binaural interaction index was within the ranges of 0.8–1.2 (Figures 2–4, bottom row). Due to low number of spikes sometimes evoked by the stimuli in the binaural stimulus matrix, if both the binaural responses to a binaural combination (repeated 30 times each) and the monaural responses to monaural stimuli at corresponding levels (repeated 30 times each) were < 6 spikes, this binaural combination was excluded from the binaural interaction classification analysis (e.g., Figure 2A4, the stimulus combination at ILD −20 dB and ABL 20 dB; Figure 2B4, the stimulus combinations at ILD -20 dB and ABL 20 dB, ILD −10 dB and ABL 20 dB; Figure 4C4, the binaural combinations at the ILD −20 dB and ABL 20 dB, the ILD −20 dB and ABL 30 dB, the ILD −10 dB and ABL 20 dB). The binaural interaction type of a neuron was then classified according to the kinds of binaural interaction behavior within the binaural stimulus matrix (Figures 2–4). To reduce the number of binaural interaction categories for a neuron, designation of no interaction (N) was not included in the binaural interaction classification scheme unless no other type of binaural interaction occurred in the matrix. The binaural interaction type of a neuron was considered to be F, I, or N if it demonstrated predominately facilitatory (F), inhibitory (I), or completely no binaural interaction (N) in the binaural stimulus matrix; the binaural response type was classified as mixed (M) if both facilitatory and inhibitory binaural interactions occurred within the binaural stimulus matrix (Figures 2–4, bottom row). Therefore, the binaural interaction type of an AI neuron was designated into one of the following: EE/F, EE/I, EE/N, EE/M, EO/F, EO/I, EO/N, EO/M, OE/F, OE/I, OE/N, OE/M, and PB.

**FIGURE 5:**
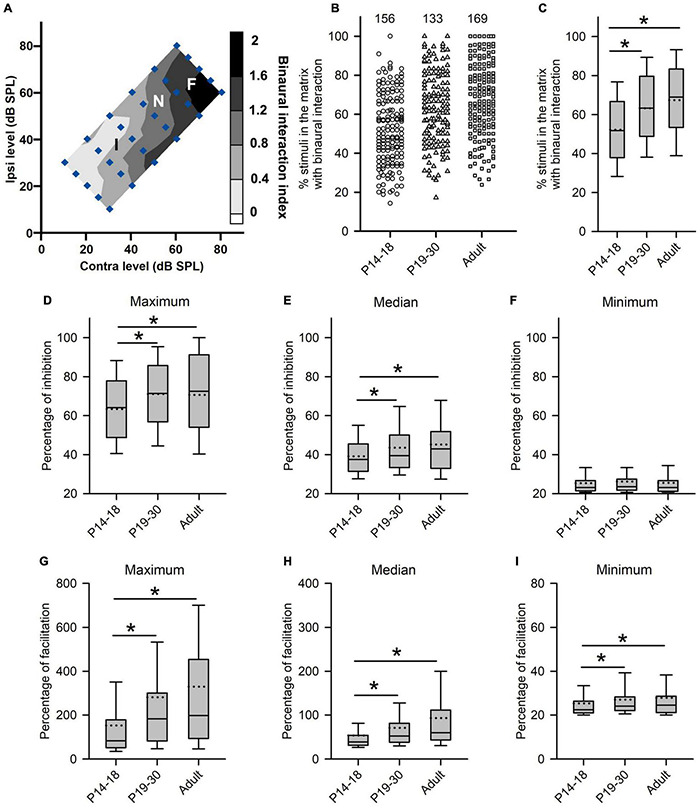
Age-related changes in the degree of binaural interactions of rat AI neurons. **(A)** Contour plot of the binaural interaction index of a representative AI neuron in responding to the binaural stimulus matrix. F, I, and N: facilitatory, inhibitory, and no binaural interaction evoked by a binaural stimulus in the matrix, respectively. **(B,C)** The scatter plot **(B)** and box plot **(C)** showing the distributions in the percentages of stimuli that evoked binaural interaction in the binaural matrix. Each symbol in panel B represents the data from one neuron. The numbers indicate the number of neurons in each group. **(D–F)** Box plots showing age-related changes in the degree of inhibitory binaural interaction. The population data for the percentages of inhibition in maximum **(D)**, median **(E)**, and minimum **(F)** are determined from each AI neuron in each group of rats. **(G–I)** Box plots showing the comparison in the degree of facilitatory binaural interaction among the three groups of AI neurons. The population data for the percentages of facilitation in maximum **(G)**, media **(H)**, and minimum **(I)** are determined from each AI neuron in each group of rats. Box plots indicate the median (solid line in the boxes), mean (dotted line in the boxes), quartiles (box extremities), and 10th/90th percentiles (error bars). * indicates significant difference between two groups (Mann–Whitney *U*-test, *p* < 0.05).

For each AI neuron, the degree of binaural interaction was determined by three aspects of analysis (Figure 5): (1) the percentage of stimuli in the binaural matrix that evoked binaural interactions, i.e., [the total number of stimulus points that evoked binaural interactions (I and F) in the binaural matrix]/[the total number of stimulus points with binaural interaction assessment (I, F, and N) in the binaural matrix] × 100%; (2) the percentage of inhibition for each inhibitory binaural interaction in the binaural matrix. This was calculated from the binaural response to a binaural stimulus and the monaural responses to monaural stimuli at corresponding monaural levels, i.e., (the sum of the monaural responses − the binaural response)/(the sum of monaural responses) × 100%. For each AI neuron, we then analyzed the maximum, median, mean, and minimum among the percentages of inhibition in the binaural matrix. (3) The percentage of facilitation for each facilitatory binaural interaction, i.e., (the binaural response to a binaural stimulus - the sum of the monaural responses at corresponding monaural levels)/(the sum of the monaural responses) × 100%. We then analyzed the maximum, median, mean, and minimum among the percentages of facilitation in the binaural matrix for each AI neuron.

Statistical analyses were performed in SPSS, and a criterion of *p* < 0.05 was considered as significantly different between groups.

## Results

The neuronal responses to both monaural stimuli and binaural stimuli were collected from 601 neurons in the AI of four groups of rats. We analyzed the population data from AI neurons in the three age groups of rats with normal hearing development [i.e., the P14–18 group (*n* = 156), the P19–30 group (*n* = 136), and the adult group (*n* = 171)] to investigate the developmental changes of the monaural response types and the binaural processing of AI neurons. We then compared the data of AI neurons between the RUCHL group (*n* = 138) and the adult group to investigate the effects of RUCHL at young age on the monaural response types and the binaural processing of AI neurons in adulthood.

Based on the monaural response properties of the 601 neurons collected in the rat AI, we classified the monaural response types of these neurons as EO, EE, OE, and PB (see example neurons in Figures 2–4). The monaural and binaural responses of the three EO neurons shown in Figure 2 demonstrated various binaural interactions. Neuron A showed predominantly facilitatory binaural interaction whereas neuron B showed predominantly inhibitory binaural interaction in the binaural stimulus matrix. Therefore, the two neurons were categorized as EO/F (Figure 2A4) and EO/I (Figure 2B4), respectively. Neuron C was categorized as EO/M because it showed predominantly facilitatory binaural interaction at ILDs + 10 to + 20 dB, and predominantly inhibitory binaural interaction at ILDs −20 to −10 dB (Figure 2C4). Figure 3 shows the responses of three EE neurons to both monaural and binaural stimuli. These EE neurons responded to monaural stimulation at either ear (Figures 3A1–C1). Their binaural responses varied with binaural stimuli in the binaural matrix (Figures 3A2–C2, A3–C3). According to the monaural response type and the binaural interaction behavior in the binaural matrix, the three EE neurons were categorized as EE/F (Figure 3A4), EE/I (Figure 3B4), and EE/M (Figure 3C4), respectively. Figure 4 shows the responses of two OE neurons (Figure 4, the first and the second columns) and one PB neuron (Figure 4, the third column) to both monaural stimuli and binaural stimuli. The two OE neurons were classified as OE/F (Figure 4A4) and OE/M (Figure 4B4). The neuron C in Figure 4 exhibited the property of predominant binaural response. By definition, this neuron was categorized as PB (Figure 4C4).

### Developmental Refinement of Binaural Processing in Primary Auditory Cortex After Hearing Onset

**FIGURE 6:**
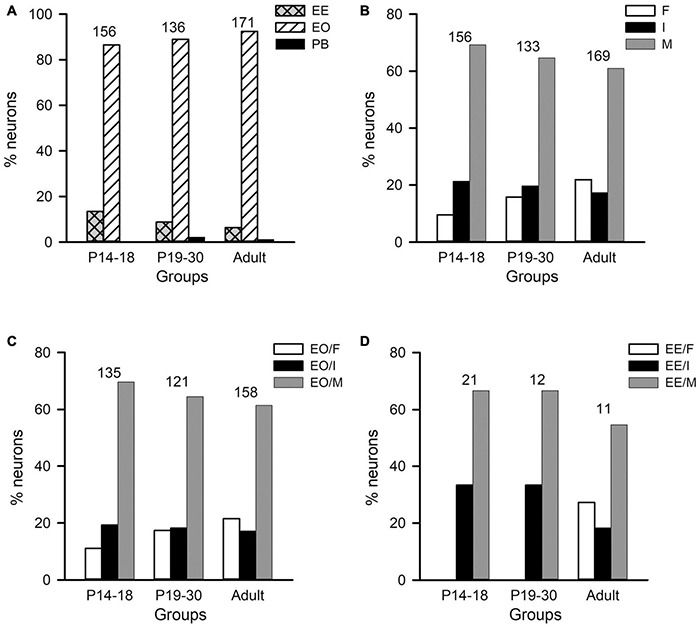
Distributions of the monaural response types and the binaural interaction types of AI neurons in the three age groups of rats. The three age groups are P14–18 group, P19–30 group, and adult group, respectively. **(A)** The distributions of the monaural response types (i.e., EE, EO, and PB) of AI neurons. **(B)** The distributions of the binaural interaction types for the population including both EO and EE neurons. The binaural interaction of each neuron was classified into one of the three categories: inhibitory (I), facilitatory (F), and mixed (M). **(C,D)** The distributions of the binaural interaction types in the population of EO neurons **(C)** and EE neurons **(D)**. Numbers shown on the top of the bars indicate the number of neurons in each age group.

We first analyzed the age-related changes in both the monaural response type and the binaural interaction type of AI neurons in the three age groups of rats with normal hearing development. The proportions of neurons within each monaural response type were as follows (Figure 6A): the P14–18 group, 86.54% for EO and 13.46% for EE; the P19–30 group, 88.97% for EO, 8.82% for EE, and 2.21% for PB; the adult group, 92.40% for EO, 6.43% for EE, and 1.17% for PB. In each age group, the proportion of EO neurons was the largest, and most neurons were categorized as EO and EE types. Because the proportions of PB neurons are very small in the three age groups, in the following data analysis, we focus on the age-related changes of binaural processing in the population of EO and EE neurons.

The population data analysis from AI neurons including both EO and EE types showed no significant differences in the distributions of binaural interaction types among the three age groups (Figures 6B–D). The majority AI neurons were categorized as mixed binaural interaction type (Figure 6B), i.e., 69.23% in the P14–18 group, 64.66% in the P19–30 group, and 60.95% in the adult group, respectively. The proportions of neurons categorized as inhibitory binaural interaction type were 21.25% in the P14–18 group, 19.55% in the P19–30 group, and 17.16% in the adult group, respectively. In addition, the proportions of neurons categorized as facilitatory binaural interaction type were 9.62% in the P14–18 group, 15.79% in the P19–30 group, and 21.89% in the adult group, respectively. We did not find significant differences in the distributions of binaural interaction types among the three age groups of A1 neurons (χ^2^-test, *df* = 4, χ^2^ = 9.219, *p* = 0.056). Within the population of EO neurons in each age group, the proportions of neurons with various binaural interaction types were as follows: the P14–18 group, 11.11% for EO/F, 19.26% for EO/I, and 69.63% for EO/M; the P19–30 group, 17.36% for EO/F, 18.18% for EO/I, and 64.46% for EO/M; the adult group, 21.52% for EO/F, 17.09% for EO/I, and 61.39% for EO/M, respectively (Figure 6C). Chi-square test did not show significant differences in the distributions of binaural interaction types among the three age groups of EO neurons (*df* = 4, χ^2^ = 5.641, *p* = 0.228). Moreover, the proportions of EE neurons with various binaural interaction types in each age group were as follows: the P14–18 group, 33.33% for EE/I and 66.67% for EE/M; the P19–30 group, 33.33% for EE/I and 66.67% for EE/M; the adult group, 27.27% for EE/F, 18.18% for EE/I, and 54.54% for EE/M (Figure 6D). We did not encounter EE/F type in the AI in both the P14–18 group and the P19–30 group. No significant differences were found in the distributions of EE/I and EE/M types among the three age groups of EE neurons (χ^2^-test, *df* = 2, χ^2^ = 0.207, *p* = 0.902).

We next determined whether there are age-related changes in the degree of binaural interactions in AI after hearing onset. One measure for the degree of binaural interaction was the percentages of the number of stimuli that evoked binaural interactions in the binaural stimulus matrix. A greater percentage from this measure implies a higher degree of binaural interaction. For each neuron, we determined the number of stimulus points with facilitatory (F), inhibitory (I), or no interactions (N) in the binaural stimulus matrix according to the binaural interaction index (Figure 5A). For the example neuron in Figure 5A, the numbers of stimulus points with facilitatory, inhibitory, or no binaural interactions were 10, 15, and 5, respectively. Consequentially, the percentage of stimulus points with binaural interactions in the binaural stimulus matrix was 83.33% (25/30) (see texts in the Methods section for detailed calculations in this measure). The population data analysis showed age-related changes in the percentages of stimuli with binaural interactions in the binaural stimulus matrix. The degree of binaural interactions in this measure was lowest in the P14–18 group than in both the P19–30 group and the adult group, and was very similar between the P19–30 group and the adult group (Figures 5B,C, Kruskal–Wallis test, χ^2^ = 47.111, *df* = 2, *p* < 0.001; Mann–Whitney *U*-test, P14–18 group vs. P19–30 group, *z* = −4.463, *p* < 0.001; P14–18 group vs. adult group, *z* = −6.638, *p* < 0.001; P19–30 group vs. adult group, *z* = −1.867, *p* = 0.062). These data demonstrated a developmental increase in the degree of binaural interaction at early postnatal age after hearing onset.

Another measure for the degree of binaural interaction is the degree of inhibition (or facilitation) when a binaural stimulus evoked the inhibitory (or facilitatory) binaural interaction. The detail method for this analysis is introduced in the materials and methods. For each AI neuron, we determined the maximum, median, and minimum of the percentage of inhibition (or facilitation) in the binaural matrix. If only one stimulus in the binaural matrix evoked an inhibitory (or facilitatory) interaction, the data were excluded from the degree of inhibition (or facilitation) analysis. For the neuron in Figure 5A, the maximum, median, and minimum percentages of inhibition in the whole binaural matrix were 91.67, 53.66, and 25.00%, respectively. In addition, the maximum, median, and minimum percentages of facilitation in the whole binaural matrix were 188.89, 42.48, 62.97, and 24.00%, respectively. The population data analysis for this measure indicates that the maximum percentages of inhibition were significantly smaller in the P14–18 group than in both the P19–30 group and the adult group; in contrast, no significant differences in the maximum percentages of inhibition were found between the P19–30 group and the adult group (Figure 5D, Kruskal–Wallis test, χ^2^ = 11.914, *df* = 2, *p* = 0.003; Mann–Whitney *U*-test, P14–18 group vs. P19–30 group, *z* = −3.023, *p* = 0.003; P14–18 group vs. adult group, *z* = −2.897, *p* = 0.004; P19–30 group vs. adult group, *z* = −0.208, *p* = 0.835). A similar trend was found in the median percentages of inhibition among groups (Figure 5E, Kruskal–Wallis test, χ^2^ = 9.949, *df* = 2, *p* = 0.007; Mann–Whitney *U*-test, P14–18 group vs. P19–30 group, *z* = −2.276, *p* = 0.023; P14–18 group vs. adult group, *z* = −2.960, *p* = 0.003; P19–30 group vs. adult group, *z* = −0.30, *p* = 0.976). However, no significant differences in the minimum percentages of inhibition were found among the three age groups of AI neurons (Figure 5F, Kruskal–Wallis test, *df* = 2, χ^2^ = 1.2149, *p* = 0.545). Using the same idea, we compared the maximum, median, and minimum percentages of facilitation in the binaural stimulus matrix among the three age groups of AI neurons. We found that the maximum percentages of facilitation were significantly smaller in the P14–18 group than in both the P19–30 group and the adult group; however, no significant differences were found in the maximum percentages of facilitation between the P19–30 group and the adult group (Figure 5G, Kruskal–Wallis test, χ^2^ = 34.397, *df* = 2, *p* < 0.001; Mann–Whitney *U*-test, P14–18 group vs. P19–30 group, *z* = −4.390, *p* < 0.001; P14–18 group vs. adult group, *z* = −5.483, *p* < 0.001; P19–30 group vs. adult group, *z* = −1.249, *p* = 0.212). A similar trend was found for the median and minimum percentages of facilitation when the population data of AI neurons were compared among the three age groups (Figure 5H, for the median percentage of facilitation, Kruskal–Wallis test, χ^2^ = 40.672, *df* = 2, *p* < 0.001; Mann–Whitney *U*-test, P14–18 group vs. P19–30 group, *z* = −4.373, *p* < 0.001; P14–18 group vs. adult group, *z* = −6.101, *p* < 0.001; P19–30 group vs. adult group, *z* = −1.883, *p* = 0.060; Figure 5I, for the minimum percentage of facilitation, Kruskal–Wallis test, χ^2^ = 10.462, *df* = 2, *p* = 0.005; Mann–Whitney *U*-test, P14–18 group vs. P19–30 group, *z* = −2.324, *p* = 0.020; P14–18 group vs. adult group, *z* = −3.066, *p* = 0.002; P19–30 group vs. adult group, *z* = −0.800, *p* = 0.424). The data in Figure 5 demonstrate that rat AI neurons undergo refinement in the degree of binaural interactions during early postnatal hearing development after hearing onset.

**FIGURE 7:**
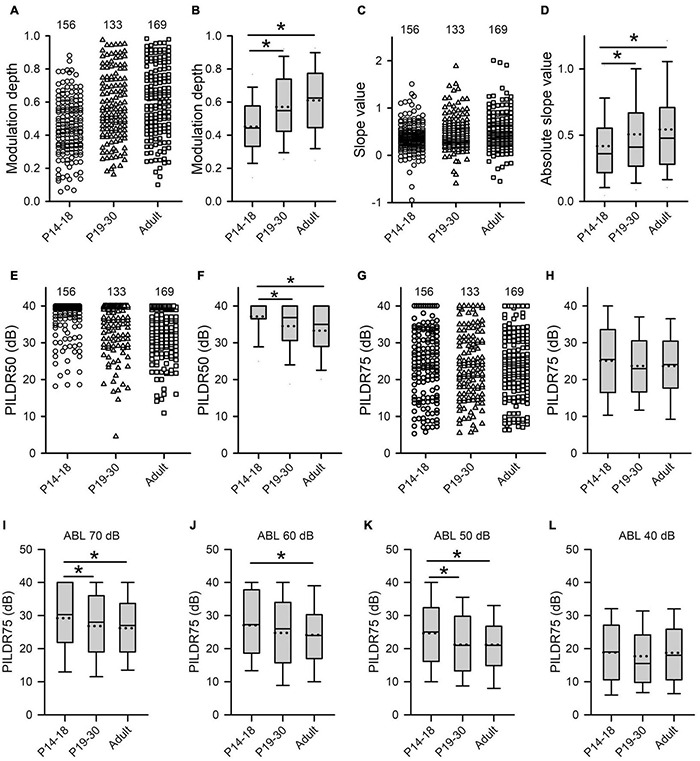
Age-related changes in both the sensitivity and the selectivity of AI neurons to ILDs determined from the ILD response functions in the three age groups. **(A–H)** Population data showing the distributions of modulation depth **(A,B)**, slope value **(C,D)**, PILDR50 **(E,F)**, and PILDR75 **(G,H)** in both scatter plots and box plots, respectively. In the scatter plots, each symbol represents the data from one neuron. The PILDR50 and the PILDR75 data shown in the second row are determined from the averaged ILD response functions across ABL. **(I–L)** Box plots showing the distributions of PILDR75 determined from the ILD response functions at ABL 40–70 dB. * indicates significant difference between two groups (Mann–Whitney *U*-test, *p* < 0.05). The greater values in the modulation depth and the absolute slope value indicate greater sensitivity to ILDs. Moreover, the smaller values in the PILDR50 and the PILDR75 indicate greater selectivity to ILDs.

To further determine the age-related changes of binaural processing after hearing onset, for each AI neuron, we determined the sensitivity of the neuron to ILD by measuring the modulation depth and the slope value from the averaged ILD response functions (see Figure 1 for methods). A greater modulation depth or a greater absolute slope value indicates a greater sensitivity to the variations of ILDs. The population data analysis showed that the values of the modulation depth were significantly smaller in the P14–18 group than in both the P19–30 group and the adult group; however, no significant differences in the modulation depth were found between the P19–30 group and the adult group (Figures 7A,B, Kruskal–Wallis test, χ^2^ = 46.927, *df* = 2, *p* < 0.001; Mann–Whitney *U*-test, P14–18 group vs. P19–30 group, *z* = −4.643, *p* < 0.001; P14–18 group vs. adult group, *z* = −6.655, *p* < 0.001; P19–30 group vs. adult group, *z* = −1.676, *p* = 0.094). The distributions of the slope values in the three groups of AI neurons indicated that the absolute slope values in the P14–18 group were smaller than those in both the P19–30 group and the adult group, and that no significant differences in the absolute slope values were found between the P19–30 group and the adult group (Figures 7C,D, Kruskal–Wallis test, χ^2^ = 11.214, *df* = 2, *p* < 0.001; Mann–Whitney *U*-test, P14–18 group vs. P19–30 group, *z* = −2.068, *p* = 0.0391; P14–18 group vs. adult group, *z* = −3.306, *p* = 0.001; P19–30 group vs. adult group, *z* = −1.676, *p* = 0.094). These data demonstrated a developmental refinement in the ILD sensitivity of AI neurons after hearing onset.

The selectivity of AI neurons to ILD was determined from the two measures: PILDR50 and PILDR75 (see methods in Figure 1). A smaller PILDR value indicates a greater selectivity for ILD. The data analysis showed that the values of PILDR50s in the P14–18 group were greater than those in both the P19–30 group and the adult group (Figures 7E,F, Kruskal–Wallis test, χ^2^ = 35.09, *df* = 2, *p* < 0.001; Mann–Whitney *U*-test, P14–18 group vs. P19–30 group, *z* = −3.8918, *p* < 0.001; P14–18 group vs. adult group, *z* = −5.845, *p* < 0.001). However, no significant differences in the values of PILDR50s were found between the P19–30 group and the adult group (Figures 7E,F, P19–30 group vs. adult group, *z* = −1.7716, *p* = 0.077). In contrast, the distributions of the PILDR75s determined from the averaged ILD response functions across ABLs in the three age groups of AI neurons were very similar (Figures 7G,H, Kruskal–Wallis test, *df* = 2, χ^2^ = 2.693, *p* = 0.260). It is possible that the PILDRs determined from the averaged ILD response functions across ABLs underestimate the ILD selectivity of the AI neurons. We further determined the PILDR75 for each neuron from the ILD response function at each ABL. The data analysis for PILDR75s at each ABL within 40–70 dB indicated that the PILDR75s of AI neurons were significantly larger in the P14–18 group than in the adult group at ABLs 50–70 dB, but not at ABL 40 dB (Figures 7I–L, Mann–Whitney *U*-test,P14–18 group vs. adult group, at ABL 70 dB, *z* = −3.034, *p* = 0.002; at ABL 60 dB, *z* = −2.598, *p* = 0.009; at ABL 50 dB, *z* = −3.004, *p* = 0.002; at ABL 40 dB, *z* = −0.120, *p* = 0.905). Moreover, the PILDR75s were significant larger in the P14–18 group than those in the P19–30 group at ABL 50 and ABL 70 dB, but not at ABL 40 dB and ABL 60 dB (Figures 7I–L, Mann–Whitney *U*-test, P14–18 group vs. the P19–30 group, at ABL 70 dB, *z* = −2.087, *p* = 0.037; at ABL 50 dB, *z* = −2.766, *p* = 0.006; at ABL 60 dB, *z* = −1.951, *p* = 0.051; at ABL 40 dB, *z* = −1.019, *p* = 0.308). In addition, we did not find significant differences in the PILDR75s between P19–30 group and adult group at ABL 40–70 dB (Figures 7I–L, Mann–Whitney *U*-test, P19–30 group vs. adult group, at ABL 70 dB, *z* = −0.778, *p* = 0.437; at ABL 60 dB, *z* = −0.665, *p* = 0.506; at ABL 50 dB, *z* = −0.109, *p* = 0.913; at ABL 40 dB, *z* = −0.963, *p* = 0.336). We also determined the PILDR50 for each neuron from the ILD response function at each ABL. The PILDR50s were significant larger in the P14–18 group than in both the P19–30 group and the adult group at ABL 40–70 dB (Mann–Whitney *U*-test, P14–18 group vs. P19–30 group: at ABL 70 dB, *z* = −3.254, *p* = 0.001; at ABL 60 dB, *z* = −4.332, *p* < 0.001; at ABL 50 dB, *z* = −3.390, *p* = 0.001; at ABL 40 dB, *z* = −2.717, *p* = 0.007. For P14–18 group vs. adult group, at ABL 70 dB, *z* = −4.833, *p* < 0.001; at ABL 60 dB, *z* = −6.740, *p* < 0.001; at ABL 50 dB, *z* = −4.879, *p* < 0.001; at ABL 40 dB, *z* = −2.708, *p* = 0.007). We did not find significant differences in the PILDR50s between P19–30 group and adult group at ABL40–70 dB (Mann–Whitney *U*-test, at ABL 70 dB, *z* = −1.154, *p* = 0.248; at ABL 60 dB, *z* = −1.729, *p* = 0.084; at ABL 50 dB, *z* = −1.3019, *p* = 0.193; at ABL 40 dB, *z* = −0.057, *p* = 0.955). These results demonstrated a developmental refinement in the selectivity of AI neurons to ILD after hearing onset.

**FIGURE 8:**
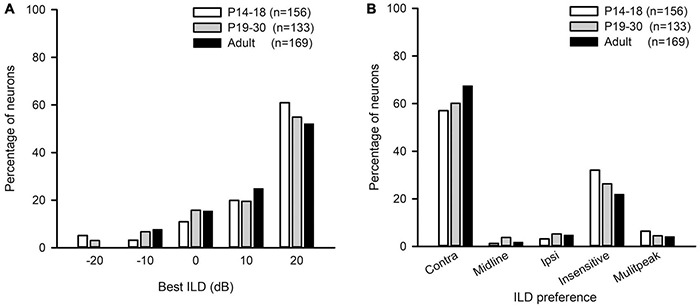
The distributions of the best ILDs and the ILD preferences of AI neurons in the three age groups of rats. **(A)** The distributions of the best ILD. **(B)** The distributions of the ILD preference. “*n*” indicates the number of neurons in each group.

The best ILD of AI neurons in the three age groups mainly distributed at ILDs 0 dB, 10 dB, and 20 dB, and only few neurons had their best ILDs at −10 dB and −20 dB (Figure 8A). We did not find significant differences in the distribution of the best ILDs of AI neurons among the three age groups (from ILD −10 to + 20 dB, χ^2^-test, *df* = 6, χ^2^ = 6.91, *p* = 0.329). We classified the ILD preference of AI neurons from the averaged ILD response functions into the following categories: contra, midline, ipsi, insensitive, and multipeak. The ILD preference was considered as “contra” if the best ILD was at + 10 dB or + 20 dB, and the PILDR75 was restrictively or predominantly distributed within the range of 0 to + 20 dB. The neuron was classified as “ipsi” ILD preference if the best ILD was at −10 dB or −20 dB, and the PILDR75 was restrictively or predominantly distributed within the range of 0 to −20 dB. The neuron was assigned as “midline” ILD preference if the best ILD was at 0 dB and the PILDR75 was restrictively distributed within the range of −10 to + 10 dB. The ILD preference was considered as “insensitive” if the continuous width of the PILDR75 was greater than 30 dB. The neuron was classified as “multipeak” if there were two or more separated PILDR75. The distributions in the ILD preferences of AI neurons in each age group demonstrate that most neurons preferred contralateral ILDs, and only few neurons preferred ipsilateral ILDs or midline ILDs (Figure 8B). With increasing ages, the AI neurons with contralateral ILD preference showed a weak trend of increase in percentages, and the largest difference in the percentages was 10.39% between the P14–18 group and the adult group; in contrast, the AI neurons that were insensitive to ILD showed a weak trend of decrease in percentages with increasing ages, and the largest difference in the percentages was 10.16% between the P14–18 group and the adult group (Figure 8B). However, no significant differences were found in the distribution of ILD preferences among the three age groups (Figure 8B, χ^2^-test, *df* = 8, *p* = 0.379).

### The Effect of Reversible Unilateral Conductive Hearing Loss at Young Age on Binaural Processing of Primary Auditory Cortex Neurons in Adulthood

**FIGURE 9:**
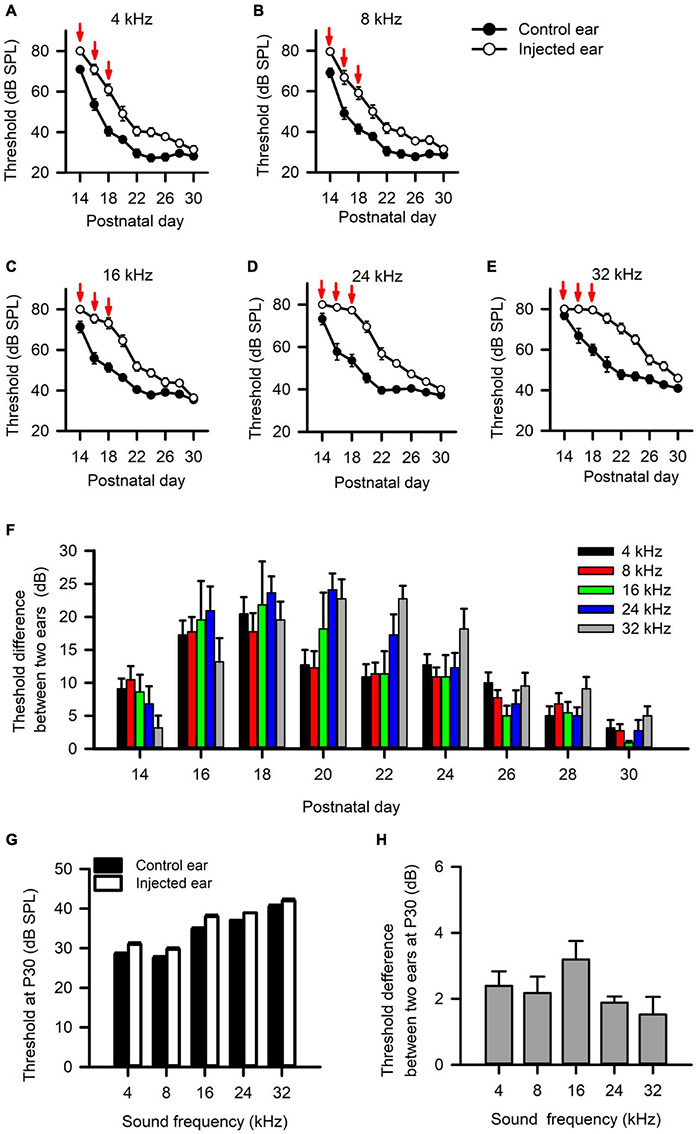
The effects of intratympanic poloxamer injections on the hearing threshold of the injected ear determined from the wave I of auditory brainstem response (ABR). **(A–E)** The response thresholds for ABR wave I elicited with tone bursts at the frequencies of 4 kHz **(A)**, 8 kHz **(B)**, 16 kHz **(C)**, 24 kHz **(D)**, and 32 kHz **(E)**, respectively. The intratympanic poloxamer injections (red arrows) were made on P14, P16, and P18 in the right ear (the injected ear). The control ear was the intact left ear. **(F)** The differences in ABR wave I threshold, i.e., the threshold of the injected ear minus the threshold of the control ear. These data are determined from 11 rats. **(G)** The ABR wave I thresholds for both the injected ear and the control ear determined at P30 (*n* = 60 rats). **(H)** The differences in ABR wave I threshold between the injected ear and the control ear determined at P30 (*n* = 60 rats).

We injected a thermoreversible poloxamer hydrogel into the middle ear cavity of one ear in rats on P14, and did additional injections at both P16 and P18 to induce RUCHL at young age. We tracked the changes of hearing thresholds determined from both the injected ear and the control ear (non-injected ear) based on the ABR wave I thresholds in a portion of rats (*n* = 11) with a 2-day interval. The data in Figure 9 indicate that intratympanic poloxamer injections elevated the ABR wave I thresholds at different tested frequencies, and the threshold differences between the injected ear and the control ear varied with postnatal days after poloxamer injection (Figures 9A–F, Wilcoxon signed-rank test, injected ear vs. control ear, *p* < 0.05 at all tested frequencies on each day). The average threshold differences were within the range of 5–25 dB until P28 (Figure 9F). At P30, we determined the ABR wave I thresholds at both the control ear and the injected ear for the 60 rats in the RUCHL group. Although the ABR thresholds at the injected ear were still higher than those at the control ear (Figure 9G, Wilcoxon signed-rank test, injected ear vs. control ear, *p* < 0.05 at all tested frequencies), the ABR threshold differences between the injected ear and the control ear at P30 were less than 5 dB (Figure 9H). We consider that the hearing thresholds at the injected ear already recovered to normal hearing at P30.

**FIGURE 10:**
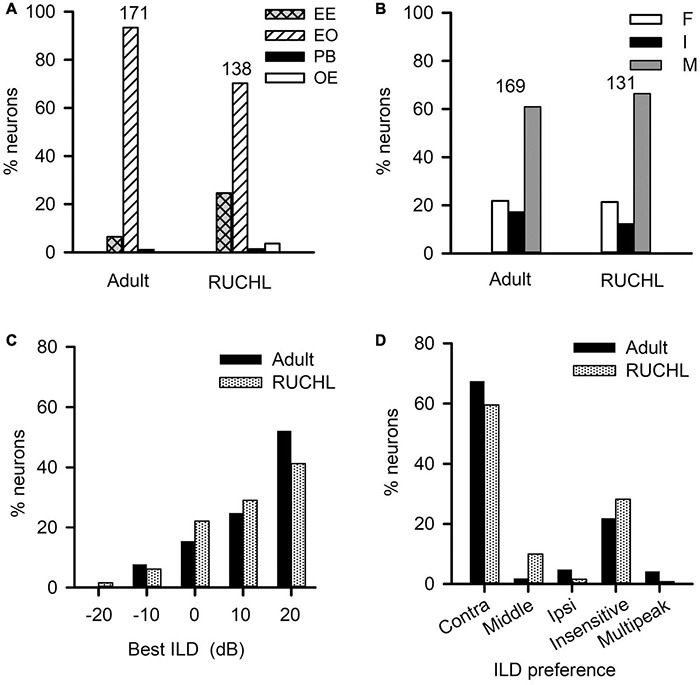
The effects of reversible unilateral conductive hearing loss (RUCHL) at young age on the monaural response types and the binaural processing of rat AI neurons in adulthood. Data from the adult group were used as control for comparison. **(A)** The distributions in the monaural response types (i.e., EE, EO, OE, and PB). **(B)** The distributions of the binaural interaction types of AI neurons including both EE and EO neurons. The binaural interaction of each neuron was classified into one of the three categories: inhibitory (I), facilitatory (F), and mixed (M). Numbers shown on the top of the bars in panels A and B indicate the number of neurons in each group. **(C)** The distribution of the best ILDs of AI neurons. **(D)** The distributions of ILD preferences of AI neurons.

To determine the effects of RUCHL at young age on the monaural response type and the binaural processing of AI neurons in adulthood, we used the data from the adult group as control. We found that RUCHL at young age decreased the proportion of EO neuron but increased the proportion of EE neurons in the AI of RUCHL rats in adulthood (Figure 10A, RUCHL group, 70.29% for EO type and 24.64% for EE type; adult group, 93.40% for EO type and 6.43% for EE type; χ^2^-test, RUCHL group vs. adult group, χ^2^ = 20.387, *df* = 1, *p* < 0.001). In addition, we encountered few OE neurons in the RUCHL group but not in the adult group (Figure 10A). Because the proportions of OE neurons (3.63%) and PB neurons (1.45%) in the RUCHL group are very small, in the following data analysis, we only focus on the data from the EO and EE neurons.

For the population of AI neurons including both EO and EE neuron in the RUCHL group (*n* = 131) and in the adult group (*n* = 169), the data showed that RUCHL at young age did not significantly change the proportions of AI neurons within each binaural interaction category or within each type of ILD preference in adulthood. The proportions of neurons categorized into different binaural interaction types are as follows (Figure 10B): RUCHL group versus adult group, 66.41% vs. 60.95% for mixed binaural interaction type, 21.37% vs. 21.89% for facilitatory binaural interaction type, and 12.21% vs. 17.16% for inhibitory binaural interaction type. No significant differences are found in the distributions of various binaural interaction types between the RUCHL group and the adult group (χ^2^-test, χ^2^ = 1.561, *df* = 2, *p* = 0.458). Similar to the distributions in the best ILDs of AI neurons in the adult group, the best ILDs of majority AI neurons in the RUCHL group are distributed in the contralateral ILDs (i.e., + 10 dB and + 20 dB), and RUCHL at young age did not significantly affect the distributions of the best ILDs of AI neurons in adulthood (Figure 10C, Fisher’s exact test, RUCHL vs. adult, *p* = 0.134). For the ILD preference, majority of AI neurons in both the RUCHL group and the adult group showed contralateral ILD preference or insensitive to the change of ILD. Fisher’s exact test showed a significant difference in the distributions of ILD preferences between the adult group and the RUCHL group (Figure 10D, *p* = 0.002). However, the proportions of AI neurons within the categories of both “contra” and “insensitive” in the RUCHL group were not significantly different from those in the adult group, respectively (Figure 10D, χ^2^-test, RUCHL vs. adult, χ^2^ = 1.913, *df* = 1, *p* = 0.167).

**FIGURE 11:**
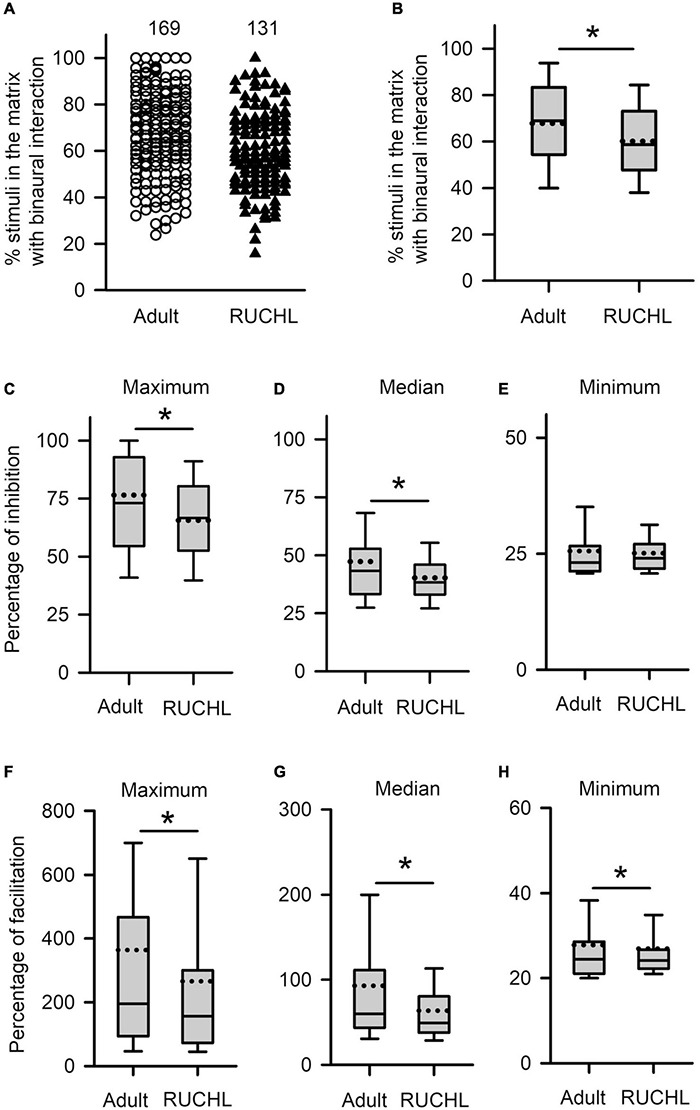
The effects of RUCHL at young age on the degree of binaural interactions of AI neurons in adulthood. **(A,B)** Scatter plot **(A)** and box plot **(B)** showing the distributions in the percentages of stimuli that evoked binaural interactions in the binaural matrix. Each symbol in panel A represents the data from one neuron. The numbers indicate the number of neurons in each group. **(C–E)** Box plots showing the comparison in the degree of inhibitory binaural interaction between the two groups of AI neurons. The population data for the percentages of inhibition in maximum **(C)**, media **(D)**, and minimum **(E)** are determined from each AI neuron in each group of rats. **(F–H)** Box plots showing the comparison in the degree of facilitatory binaural interaction between the two groups of AI neurons. The population data for the percentages of facilitation in maximum **(F)**, media **(G)**, and minimum **(H)** are determined from each AI neuron in each group of rats. * indicates significant difference between two groups (Mann–Whitney *U*-test, *p* < 0.05).

We next determined the effects of RUCHL at young age on the degrees in binaural interactions of AI neurons in adulthood. The data in Figure 11 demonstrated that RUCHL at young age decreased the degree of binaural interactions of AI neurons in adulthood. We used Mann–Whitney *U*-test to compare the data between the RUCHL group and the adult group, and found that the percentages in the number of stimuli evoking binaural interactions in the binaural matrix were significantly lower in the AI neurons of the RUCHL group than in the AI neurons of the adult group (Figures 11A,B, RUCHL vs. adult, *z* = −3.585, *p* < 0.001). We also found that, for the population of AI neurons, the maximum and the median percentages of inhibition in the binaural matrix in the RUCHL group were significantly smaller than those in the adult group, respectively (Figure 11C, maximum, *z* = −2.007, *p* = 0.045; Figure 11D, median, *z* = −2.059, *p* = 0.040). However, no significant differences in the minimum percentages of inhibition were found between the RUCHL group and the adult group (Figure 11E, *z* = −1.087, *p* = 0.277). In addition, the maximum, median, and minimum percentages of facilitation in the binaural matrix for the AI neurons in the RUCHL group were significantly smaller than those in the adult group, respectively (Figure 11F, maximum, *z* = −2.009, *p* = 0.045; Figure 11G, median, *z* = −3.140, *p* = 0.002; Figure 11H, minimum, *z* = −2.099, *p* = 0.036).

**FIGURE 12:**
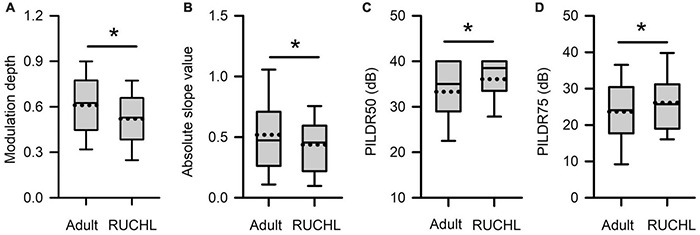
Box plots showing the effects of RUCHL at young age on the sensitivity and the selectivity to ILDs of AI neurons in adulthood. **(A)** Modulation depth; **(B)** absolute slope value; **(C)** PILDR50; **(D)** PILDR75. The data for the PILDR50 the PILDR75 were determined from the averaged ILD response functions across ABL. * indicates significant difference between two groups (Mann–Whitney *U*-test, *p* < 0.05).

To determine whether RUCHL at young age affects the tuning of AI neurons to ILDs in adulthood, we compared both the sensitivity and the selectivity of AI neurons to ILDs between the RUCHL group and the adult group by Mann–Whitney *U*-test. The results demonstrated that RUCHL at young age decreased both the selectivity and the sensitivity of AI neurons to ILDs in adulthood. For the sensitivity of AI neurons to ILDs, both the values of the modulation depth and the absolute slope values of the averaged ILD response functions were significantly smaller in the RUCHL group than in the adult group, respectively (Figure 12A, *z* = −3.576, *p* < 0.001; Figure 12B, *z* = −1.988, *p* = 0.047). For the selectivity of AI neurons to ILDs, our data showed that the preferred ILD ranges of AI neurons determined from the averaged ILD response functions were significantly larger in the RUCHL group than in the adult group (Figure 12C, PILDR50, *z* = −2.985, *p* = 0.003; Figure 12D, PILDR75, *z* = −2.090, *p* = 0.037). We further analyzed the PILDR50 and PILDR75 of AI neurons determined from the ILD response functions at ABLs 40–70 dB. We found that the PILDR75s of AI neurons were larger in the RUCHL group than in the adult group at ABLs 50–70 dB but not at ABL 40 dB (RUCHL group vs. adult group, at ABL 70 dB, *z* = −3.029, *p* = 0.002; at ABL 60 dB, *z* = −3.029, *p* = 0.002; at ABL 50 dB, *z* = −2.994, *p* = 0.003; at ABL 40 dB, *z* = −1.674, *p* = 0.094). Moreover, the PILDR50s of AI neurons were larger in the RUCHL group than in the adult group at ABLs 50–70 dB but not at ABL 40 dB (RUCHL group vs. adult group, at ABL 70 dB, *z* = −4.529, *p* < 0.001; at ABL 60 dB, *z* = −4.642, *p* < 0.001; at ABL 50 dB, *z* = −2.322, *p* = 0.020; at ABL 40 dB, *z* = −1.359, *p* = 0.174).

### Comparison of the Binaural Processing of Primary Auditory Cortex Neurons Between Reversible Unilateral Conductive Hearing Loss Rats and Immature Rats

To determine whether the RUCHL halt the development of binaural processing, we compared the data from the RUCHL rats and the data from two groups of immature rats by Kruskal–Wallis test and Mann–Whitney *U*-test. The results indicate that, to some extent, brief RUCHL at early age seems to retard the refinement of binaural processing at young age in the degree of binaural interaction, and in both the selectivity and sensitivity to ILDs of AI neurons.

The percentages of stimuli evoking binaural interactions in the binaural matrix were higher in the RUCHL group than in the P14–18 group (*p* < 0.001), but were similar between the RUCHL group and the P19–30 group (*p* = 0.178). In addition, the maximum percentages of inhibition in the RUCHL group were similar to those in the P14–18 group (*p* = 0.476), but were lower than those in the P19–30 group (*p* = 0.049). The median (and the minimum) percentages of inhibition were not significantly different among the three groups (Kruskal–Wallis test, for the median, *p* = 0.068; for the minimum, *p* = 0.332). The maximum and the median percentages of facilitation in the RUCHL group were higher than those in the P14–18 group (both *p* = 0.001), but were not significantly different from those in the P19–30 group, respectively (for maximum, *p* = 0.346; for median, *p* = 0.246). No significant differences were found in the minimum percentages of facilitation among the three groups (Kruskal–Wallis test, *p* = 0.082).

The absolute slope values were not significantly different among the three groups (Kruskal–Wallis test, *p* = 0.100). However, the modulation depths in the RUCHL group were larger than those in the P14–18 group (*p* = 0.001) and were similar to those in the P19–30 group (*p* = 0.119). Kruskal–Wallis tests showed that the PILDR75s were not significantly different among the three groups at ABL 70 dB (*p* = 0.069), ABL 60 dB (*p* = 0.082), and ABL 40 dB (*p* = 0.061) except at ABL 50 dB (*p* = 0.007). At ABL 50, the PILDR75s in the RUCHL group were similar to those in the P14–18 group (*p* = 0.721), but were larger than those in the P19–30 group (*p* = 0.002). Moreover, the PILDR50s in the RUCHL group were significantly larger than those in the P19–30 group at ABL 70 dB (*p* = 0.002) and ABL 60 dB (*p* = 0.018), but not at ABL 50 dB (*p* = 0.426) and ABL 40 dB (*p* = 0.138); the PILDR50s in the RUCHL group were significantly smaller than those in the P14–18 group at ABL 60 dB (*p* = 0.024) and ABL 50 dB (*p* = 0.002), but not at ABL 70 dB (*p* = 0.636) and ABL 40 dB (*p* = 0.121), respectively.

### The Basic Response Properties of Primary Auditory Cortex Neurons in the Four Groups of Rats

The CFs of AI neurons in the four groups of rats are shown in Table 1. Whereas the CFs of AI neurons in the P14–18 group were significantly different from those both in the P19–30 group and in the adult group, the CFs of AI neurons showed no significant differences between the P19–30 group and the adult group (Mann–Whitney *U*-test, P14–18 group vs. P19–30 group, *z* = −3.780, *p* < 0.001; P14–18 group vs. adult group, *z* = −2.543, *p* = 0.011; P19–30 group vs. adult group, *z* = −1.059, *p* = 0.289). In addition, we did not find significant differences in the CFs of AI neurons between the RUCHL group and the adult group (Mann–Whitney *U*-test, *z* = −0.530, *p* = 0.596). For the neurons categorized into inhibitory, facilitatory, and mixed binaural interactions, we did not find specific CF bands that contributed to one of three binaural interaction categories.

**TABLE 1:** The basic response properties of primary auditory cortex (AI) neurons in the four groups of rats.

	**P14–18 group**	**P19–30 group**	**Adult group**	**RUCHL group**
CF (kHz)	21.08 ± 10.05^*#^	25.63 ± 10.69	24.06 ± 11.04	23.59 ± 11.60
MT (dB)	29.42 ± 10.24*	28.16 ± 7.81	26.60 ± 7.68^&^	28.99 ± 7.67
Latency (ms)	24.23 ± 5.31^*#^	16.73 ± 3.47^$^	15.05 ± 3.18	14.29 ± 3.54

Data are shown in mean and SD. CF, characteristic frequency; MT, minimum threshold. *, #, $, and & indicate *p* < 0.05 (Mann–Whitney *U*-test) at P14–18 group vs. adult group, P14–P18 group vs. P19–30 group, P19–30 group vs. adult group, and UCHL group vs. adult group, respectively.

We used CF stimuli to determine the minimum threshold of each neuron at 0 dB ILD (i.e., equal levels at both ears) with ABL varying from 0 to 80 dB at 10-dB steps. The minimum threshold was defined as the ABL that elicited 20% of the maximum response in the rate versus ABL function. The minimum thresholds of AI neurons in the four groups of rats are shown in Table 1. The minimum thresholds of AI neurons were higher in the P14–18 group than in the adult group; however, the minimum thresholds of AI neurons in the P19–30 group were not significantly different from those both in the P14–18 group and in the adult group (Mann–Whitney *U*-test, P14–18 group vs. adult group, *z* = −2.987, *p* = 0.003; P14–18 group vs. P19–30 group, *z* = −1.418, *p* = 0.156; P19–30 group vs. adult group, *z* = −1.647, *p* = 0.099). In addition, the minimum thresholds of AI neurons were significantly different between the RUCHL group and the adult group (Mann–Whitney *U*-test, *z* = −2.732, *p* = 0.006).

We analyzed the response latencies (i.e., first spike latencies) of AI neurons to the sound stimulus with 70 dB SPL at both ears. The response latencies of AI neurons in the four groups of rats are shown in Table 1. Mann–Whitney *U*-test showed that the response latencies were significantly different among AI neurons in the three age groups of rats with normal hearing development (P14–18 group vs. P19–30 group, *z* = −11.522, *p* < 0.001; P14–18 group vs. adult group, *z* = −13.888, *p* < 0.001; P19–30 group vs. adult group, *z* = −4.454, *p* < 0.001); however, the response latencies of AI neurons were not significantly different between the RUCHL group and the adult group (*z* = −1.866, *p* = 0.062).

Our data analysis did not find a specific range in CFs, minimum thresholds, and response latencies that contributes to a specific binaural interaction type; therefore, we did not find any specific relationships between the basic response properties and the binaural interaction types. Due to the time-consuming nature of the data collection for each neuron in our experimental design, it was difficult to get at a complete tonotopic map in one rat, and consequentially we could not determine the relationship between the basic response properties and the tonotopic maps.

## Discussion

During postnatal hearing development, the perception of sound spatial locations undergoes age-related changes (Freigang et al., 2015), experience-dependent plasticity (Keating and King, 2013; Keating et al., 2015), and training-induced plasticity in both behavior performance and auditory cortical spatial tuning (Zhang et al., 2013; Keating et al., 2016; Cheng et al., 2020). Whereas early age bilateral conductive hearing loss impairs sound loudness perception (Sun et al., 2011) and spatial memory (Zhao et al., 2018), UCHL during development impair performance on tasks such as sound localization and spatial release from masking that rely on binaural processing (Kumpik and King, 2019). The effects of hearing loss on the cognitive function depend on both the type of hearing loss and the time period of hearing loss during development. In the present study, we used CF tones to determine the age-related changes of binaural processing after the onset of hearing in the rat AI and investigated the effects of RUCHL at young age on the binaural processing of AI neurons in adulthood.

The results in the present study show that the EO and EE types are the two dominant monaural response types, and that the neurons in the rat AI exhibit inhibitory, facilitatory, and mixed binaural interactions. Our data have demonstrated that (1) the monaural response type, the binaural interaction type, and the distributions of the best ILDs in the rat AI are already adult-like shortly after hearing onset; (2) there exist developmental refinements in binaural processing, which were exhibited by an increase in the degree of binaural interaction, and the increase in the sensitivity and selectivity to ILDs during early period after hearing onset. (3) RCUHL at young age disrupts the developmental refinement of binaural processing of AI neurons in adulthood, i.e., decreases the degree of binaural interactions, and decreases both the selectivity and sensitivity to ILDs of AI neurons in adulthood. These results might help us to understand the neuronal mechanism of the refinement and plasticity in the perception of sound spatial locations during hearing development.

### Postnatal Development in the Binaural Interactions in Primary Auditory Cortex

Our data indicate that the degree of binaural interactions in rat AI is relative weak at early ages after hearing onset and then progressively strengthens to maturity. In the binaural stimulus matrix, the percentages of stimuli evoking binaural interactions increase with age during the early period after hearing onset. Whereas the degrees of inhibitory/facilitatory binaural interactions in rat AI neurons are the lowest in the P14–18 group, they showed no significant differences between the P19–30 group and the adult group (Figure 5). To our knowledge, this is the first study to determine the developmental refinements in the degree of binaural interactions of AI neurons.

The auditory responses to binaural stimuli at early period after hearing onset have been reported in several previous studies. Evoked potential studies have demonstrated that human newborn infants show detectable although immature binaural interactions in the brainstem (Cone-Wesson et al., 1997) and auditory cortex (Nemeth et al., 2015). At single neuron level, the responses of cat AI neurons are influenced by binaural stimuli as early as at P8 before the hearing thresholds have declined below 100 dB SPL, and the binaural interaction types determined by P44 were similar in kind to those recorded in adult cats (Brugge et al., 1988). The hearing onset of rats occurs normally at P12, and the sound-evoked neuronal responses with high thresholds and poor frequency selectivity could be recorded in the rat auditory cortex as early as P13 (Zhang et al., 2001). In the present study, we determined the binaural interactions of rat AI neurons using CF tones from P14 because the response thresholds and frequency selectivity of rat AI neurons at P12–13 are not suitable for us to evaluate the binaural processing in our experiment paradigm. Our results show that both the monaural response types and the binaural interaction types in the P14–18 group are similar to those in the P19–30 group and the adult group. In the auditory cortex of pallid bats, the adult-like clustered organization of binaural properties is present at P15 before the morphological development of external ears and head is complete; however, the binaural facilitation was not observed in bats younger than 25 days (Razak and Fuzessery, 2007). Therefore, previous studies from cats and bats, and the present study from rats have demonstrated that the binaural interaction types observed immediately after hearing onset are largely adult-like. Furthermore, the present study demonstrates that the degree of binaural interactions undergoes developmental increase after hearing onset.

In the adult cat AI, purely monaural neurons are rare (Zhang et al., 2004). Consistent with this result, we found that all of the recorded rat AI neurons showed some sort of binaural interactions. For those stimuli that evoked binaural interactions in the binaural stimulus matrix, the responses to the sound stimuli in one ear can be facilitated and/or suppressed by presenting the stimuli at the other ear. Even in the AI of RUCHL rats that experienced asymmetric binaural hearing, we did not encounter purely monaural neurons. Although we cannot rule out the possibility of monaural neurons in the rat AI, the data in the present study suggest that purely monaural neurons are rare in the rat AI.

### Postnatal Development of Interaural Level Differences Processing in the Auditory Cortex

The ability to perceive the acoustic space changes during postnatal hearing development (Freigang et al., 2015). In the central auditory system, the encoding of sound spatial information undergoes maturational changes after hearing onset. For example, the rudimentary ILD coding in the lateral superior olive (Sanes and Rubel, 1988), inferior colliculus (Blatchley and Brugge, 1990), and auditory cortex (Brugge et al., 1988; Mrsic-Flogel et al., 2003) is evident soon after the onset of hearing and further matures with age (Moore and Irvine, 1981). The ILD responses in the lateral superior olive of young gerbils show smaller dynamic range and shallower slope than that of adult gerbils (Sanes and Rubel, 1988). Moreover, EEG studies in 4–7 years age of normal hearing children have demonstrated that adult-like ILD coding patterns are evident in the immature cortical responses of children at this age (Easwar et al., 2018).

In the present study, we found that AI neurons in the P14–18 group had larger values in PILDRs and smaller values in the slope and the modulation depth of the ILD response functions than the AI neurons in both the P19–30 group and the adult group (Figure 7). The results indicate that both the sensitivity and the selectivity of rat AI neurons to ILD are immature during 1 week after the onset of hearing. Moreover, the sensitivity and the selectivity to ILD for AI neurons in the P19–30 group were similar to those in the adult group. Therefore, both the sensitivity and the selectivity of rat AI neurons to ILD undergo a developmental refinement after hearing onset, which might contribute to the development of auditory spatial tuning in rat AI.

The factors that influence the development of ILD processing in AI could be from auditory peripheral and/or central neural circuits. During postnatal hearing development, the binaural cues (ITD and ILD) vary as the head and the external ears grow. The auditory periphery has been demonstrated to be critically involved in limiting the maturation of spatial selectivity in the ferret auditory cortex (Mrsic-Flogel et al., 2003). In the present study, we determined the binaural responses of high-frequency (> 4 kHz) AI neurons to stimuli varying in ILDs. The age-related changes in ILDs from auditory peripheral could be one factor contributing to the observed refinement of ILD processing in AI. Previous studies have shown that binaural interaction shapes the virtual space receptive field and changes the spatial selectivity of AI neurons (Brugge et al., 1994). In the present study, the age-related changes in the degree of binaural interactions in rat AI might be another factor in the central circuits contributing to the developmental refinement of ILD processing. However, how the age-related changes in binaural interactions directly refine the ILD processing during hearing development is still not clear.

Our data show that majority of AI neurons preferred ILDs favoring contralateral spatial azimuth in both the adult and developing rats (Figure 8). Studies in the adult animals in a variety of species have demonstrated the dominant preference of AI neurons to contralateral spatial locations, e.g., in cats (Irvine et al., 1996; Zhang et al., 2004), bats (Razak, 2011), monkeys (Zhou and Wang, 2012; Lui et al., 2015), rats (Yao et al., 2013; Gao et al., 2018; Wang et al., 2019), and mice (Panniello et al., 2018). The results in the immature rats suggest that the dominant contralateral preference of AI neurons is already present immediately after the onset of hearing and is adult-like.

### The Effect of Reversible Unilateral Conductive Hearing Loss at Young Age on the Binaural Processing in Adult Primary Auditory Cortex

In the present study, we injected the poloxamer into the right ear of rats to induce a short-term UCHL in the RUCHL group. This manipulation induced an asymmetric binaural hearing because UCHL degraded the excitatory input from the injected ear whereas the excitatory input from the non-injected ear remained normal. Even if the hearing threshold of the injected ear returned to normal at P30 (Figure 9), the AI neurons in the RUCHL group showed a lower degree of binaural interaction and lower sensitivity and selectivity to ILDs compared with the AI neurons in adult group (Figure 10). We have shown the developmental refinement in the binaural processing of AI neurons during postnatal hearing development; the data from the RUCHL group indicated that the short-term asymmetric binaural hearing at young age disrupted the developmental refinement of binaural processing in the rat AI in adulthood, and the effects lasted at least 1 month after the hearing threshold of the injected ear recovered.

Reversible Unilateral Conductive Hearing Loss at young age did not significantly affect the distributions in both the ILD preferences and the best ILDs of AI neurons (Figure 10). Previous studies have shown that a stronger and longer period of UCHL (by removing the tympanic membrane and malleus in the right ear from P14) greatly reduced the proportion of rat AI neurons with contralateral azimuth preference and increased the proportion of AI neurons with ipsilateral azimuth preference when determined in adulthood (Wang et al., 2019). In addition, monaural deprivation in rats by ear canal ligation from P14 weakened the deprived ear’s representation, strengthened the open ear’s representation, and disrupted binaural integration of ILD in AI (Popescu and Polley, 2010). It is very likely that the short-term and moderate UCHL used in the present study was not strong enough to change the distributions in both the ILD preferences and the best ILD of AI neurons in the RUCHL group.

For the monaural response types in rat AI, RUCHL at young age decreased the proportion of EO neurons and increased the proportion of EE neurons in the RUCHL group; in addition, we observed few OE neurons in the RUCHL group but not in the adult group with normal hearing development (Figure 10A). Although the differences in the proportions of neurons within the same monaural response type category between the RUCHL group and the adult group are not large, the data from our study indicated that, for a small proportion of AI neurons in the RUCHL group, the RUCHL at young age indeed increased representation of ipsilateral normal hearing ear, and consequentially increased the proportions of EE neurons and OE neurons in the rat AI of RUCHL group. Furthermore, the results suggest that the adult-like distributions in monaural response types of AI neurons immediately after hearing onset can be modified by abnormal hearing experience during the early period of hearing development.

### Possible Mechanisms for the Developmental Plasticity of Binaural Processing

During postnatal hearing development, the auditory system undergoes developmental changes in synaptic responses and synaptic receptor components. The synaptic responses in the lateral superior olive of P12–14 gerbils indicate that both the amplitude and temporal processing remain compromised (Sanes, 1993). In the mouse auditory cortex, both the intrinsic and synaptic properties undergo a transitional period between P10 and P18 prior to reaching steady state at P19–P29 (Oswald and Reyes, 2008). Immediately after hearing onset, the excitatory and the inhibitory synaptic responses in rat AI to sound stimuli are equally strong; however, the excitation and inhibition are not matched and correlated until during the third postnatal week (Dorrn et al., 2010). In addition, the inhibitory synaptic inputs in developing AI are longer in duration than those in adult AI (Cai et al., 2018). The refinement of binaural processing found in the present study might depend on the changes of the inhibitory and excitatory synaptic responses in AI (Dorrn et al., 2010; Sun et al., 2010; Cai et al., 2018), or the changes of the strength of inhibitory and excitatory intracortical connections in AI (Chang et al., 2005). During postnatal hearing development, the synaptic receptor components in AI also undergo age-related changes. In the rat auditory cortex, AMPA receptor subunit GluR2 protein expression increases with age after hearing onset (Xu et al., 2007), and the expression of NMDA receptor subunit NR1 mRNA increases from birth to P35 (Lu et al., 2008). In addition, the NR2A and NR2B mRNA expression in the rat auditory cortex peaks about 1 week after the onset of hearing before declining slightly into adulthood (Hsieh et al., 2002). Moreover, the GABAA receptor subunit (α1 and α3) expression in the rat auditory cortex also exhibits age-related changes (Xu et al., 2010). These age-related changes in the subunits of AMPA, NMDA, and GABA receptors might play an important role in the auditory functional development. However, whether these changes are directly related to the developmental refinements of binaural processing observed in the present study needs to be further studied.

Acoustic experience can induce plasticity in the neuronal connections and aural dominance in the central auditory system. During the critical period of hearing development, the neuronal connections in the mouse auditory cortex are shaped by hearing experience (Meng et al., 2020). Early onset of unilateral deafening cats leads to a massive reorganization of aural preference in the auditory cortex in favor of the hearing ear (Kral et al., 2013). Moreover, UCHL in young rats induced a significant shift of the aural dominance from contralateral preference to ipsilateral preference in AI but not in the inferior colliculus (Popescu and Polley, 2010), and this result suggests that the effect of RUCHL exerts stronger plasticity in the auditory cortex than in the inferior colliculus. Consistently, a 2-deoxyglucose uptake study in gerbils demonstrated that RUCHL at young age could restore balanced afferent activity on both sides of the anteroventral cochlear nucleus, the medial superior olive, and the inferior colliculus 1 week after restoring binaural hearing from P28 (Hutson et al., 2009). Based on these findings, we postulate that the observed RUCHL effects in the present study most likely occurred above the inferior colliculus. In the present study, the RUCHL at young age did not significantly change the CF distributions and the response latencies of AI neurons in adulthood. Our data showed that, to some extent, RUCHL at young age might retard the refinement of binaural processing in the degree of binaural interaction, and in both the selectivity and sensitivity to ILDs of AI neurons. It is possible that the RUCHL at young age might induce abnormal development changes in the excitatory/inhibitory synaptic responses and the synaptic receptor components in the auditory cortex, and therefore disrupt normal developmental refinement of binaural processing.

The present study was conducted in urethane-anesthetized rats. Because the data collection in the experimental design was time consuming, it is difficult to perform this study in awaking rats. If urethane has any effects on the neuronal responses in AI, it would affect both the monaural and binaural responses. As we determined the binaural interactions of AI neurons by comparing the binaural responses with monaural responses, this possible effect should not have great influences on our conclusions.

## Data Availability Statement

The raw data supporting the conclusions of this article will be made available by the authors, without undue reservation.

## Ethics Statement

All experimental procedures were approved by the Institutional Animal Care and Use Committee (IACUC) of East China Normal University. All efforts were made to minimize the suffering of animals and the number of animals used.

## Author Contributions

JZ and JL designed this study, analyzed the data, and drafted the article. JL and XH engaged in data collection. All authors contributed to the final version of the article and approved the submitted version.

## Conflict of Interest

The authors declare that the research was conducted in the absence of any commercial or financial relationships that could be construed as a potential conflict of interest.

## Publisher’s Note

All claims expressed in this article are solely those of the authors and do not necessarily represent those of their affiliated organizations, or those of the publisher, the editors and the reviewers. Any product that may be evaluated in this article, or claim that may be made by its manufacturer, is not guaranteed or endorsed by the publisher.

## Abbreviations

UCHL: unilateral conductive hearing loss
RUCHL: reversible unilateral conductive hearing loss
AI: primary auditory cortex
ABL: average binaural level
ILD: interaural level difference
ITD: interaural time difference
CF: characteristic frequency
P: postnatal day
PILDR: preferred ILD range
PILDR75: the PILDR over which the normalized response strength was 0.75
PILDR50: the PILDR over which the normalized response strength was 0.5
ABR: auditory brainstem response.
